# Identification of QTLs controlling hydrolyzable tannin contents derived from a wild tea relative, *Camellia taliensis*

**DOI:** 10.1270/jsbbs.24079

**Published:** 2025-07-31

**Authors:** Kazuhiro Takayama, Fumiya Taniguchi, Shuya Yamashita, Akiko Ogino, Manami Monobe, Sachiko Nomura, Kaori Ema, Katsuyuki Yoshida

**Affiliations:** 1 Institute of Fruit Tree and Tea Science (NIFTS), National Agriculture and Food Research Organization (NARO), 87 Seto, Makurazaki, Kagoshima 898-0087, Japan; 2 NIFTS, NARO, 2-1 Fujimoto, Tsukuba, Ibaraki 305-8605, Japan; 3 NIFTS, NARO, 2769 Shishidoi, Kanaya, Shimada, Shizuoka 428-8501, Japan

**Keywords:** tea, theogallin, genetic map, *Camellia sinensis*

## Abstract

‘MK5601’ is an improved tea cultivar with genetic material from *Camellia taliensis*, a wild relative of the tea plant, *Camellia sinensis*. ‘MK5601’ differs from common tea cultivars in being rich in hydrolyzable tannins such as theogallin and G-strictinin, which have presumed health benefits. We identified two quantitative trait locus (QTL) clusters with powerful effects on hydrolyzable tannin contents in a population derived from a cross between ‘MK5601’ and an elite green tea cultivar ‘Saeakari’. The *C. taliensis*-type alleles at each locus in ‘MK5601’ increased the hydrolyzable tannin contents. The two QTL clusters were detected in 2 years and were confirmed in another population. These clusters have pleiotropic effects on tannin biosynthesis, as they are associated also with catechin contents. There was a strong negative correlation between the contents of hydrolyzable tannins and non-galloylated catechins. Despite the remarkable compositional changes, the two QTL clusters did not affect tea flavor. These QTLs provide important basic knowledge for developing new tea cultivars and tea products with high contents of hydrolyzable tannins and good health-promoting effects.

## Introduction

There is growing demand in Japan for the development of foods that support the health of the elderly (Maeda-Yamamoto 2017). Tea (*Camellia sinensis*) has long been known for its medicinal properties, and studies suggest that green tea consumption helps combat obesity and has a variety of other benefits, such as improving blood sugar levels, promoting bone health, and fighting cancer (Abiri *et al.* 2023). These effects are attributed mainly to epigallocatechin gallate, the major bioactive compound in green tea. Food scientists and breeders have explored other ingredients and tea cultivars to enhance the health-promoting effects and diversity of tea products. For example, ‘Benifuuki’ is rich in epigallocatechin-3-O-(3-O-methyl) gallate, which has been shown to reduce allergy, and ‘Sunrouge’ is rich in anthocyanins, which are thought to relieve eyestrain (Masuda *et al.* 2014, Saito *et al.* 2011). ‘Benifuuki’ was selected from progeny of crosses with resources from Darjeeling, and ‘Sunrouge’ from progeny of crosses with related species (Nesumi *et al.* 2012, Takeda *et al.* 1994). The tea cultivars commonly grown in Japan have been intensively selected from limited genetic resources, resulting in low genetic diversity (Kaundun *et al.* 2000), and the genetic diversity of Japanese tea germplasms is generally low (Taniguchi *et al.* 2014). To develop tea cultivars with new chemical characteristics, it is important to use genetic resources introduced from other countries and tea relatives, as exemplified by the development of ‘Benifuuki’ and ‘Sunrouge’.

**Fig. 1.**
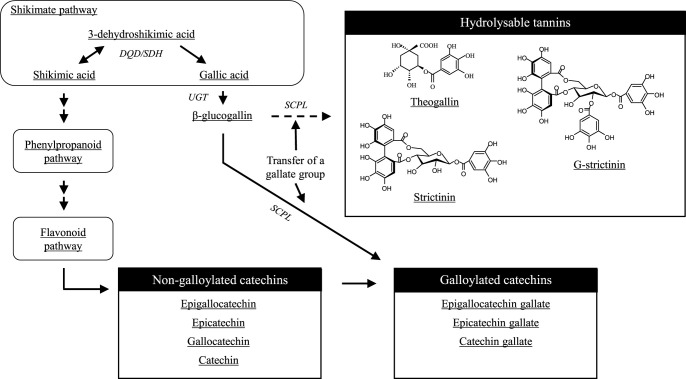
Tannin biosynthesis pathway in tea.

Tea is produced primarily from two main subspecies, ssp. *sinensis* and ssp. *assamica*, but 11 other *Camellia* species are consumed as tea within their native regions (Meng *et al.* 2019). One of these, *Camellia taliensis*, has a similar chemical composition to *C. sinensis*, being rich in flavan-3-ols and caffeine. The analysis of wild plants in multiple regions identified abundant hydrolyzable tannins considered as characteristic of *C. taliensis* (Gao *et al.* 2008, Zhu *et al.* 2012). One Japanese tea cultivar, ‘Cha Chuukanbohon Nou 6’, selected from a cross of *C. taliensis* × *C. sinensis*, is also rich in hydrolyzable tannins, especially theogallin and G-strictinin (1,2-di-O-galloyl-4,6-O-(S)-hexahydroxydiphenoyl-β-d-glucopyranose) (Honma *et al.* 2010). G-strictinin is structurally similar to strictinin, but with an additional gallate group of a d-glucose backbone (Fig. 1). ‘MK5601’, developed by crossing *C. sinensis* with ‘Cha Chuukanbohon Nou 6’, contains high levels of these hydrolyzable tannins. The biological effects of theogallin and G-strictinin have not been extensively studied, as they are present at relatively low levels in typical tea cultivars. However, the infusion of the theogallin-rich ‘MK5601’ was effective in preventing age-related changes in cognitive function in animal studies (Nomura *et al.* 2021). In addition, strictinin and G-strictinin have been reported to have anti-allergenic properties by inhibiting IgE production (Honma *et al.* 2010, Tachibana *et al.* 2001). G-strictinin has been detected in *Camellia oleifera* and walnuts (Fukuda *et al.* 2003, Yoshida *et al.* 1994), but it is absent in most *C. sinensis* cultivars, except for the Kenyan cultivar ‘TRFK306’, which is known as purple tea (Shimoda *et al.* 2015). Consumption of purple tea extract containing G-strictinin improved exercise performance, decreasing lactate dehydrogenase levels in humans, and improving lower-body muscle endurance (Cesareo *et al.* 2020). Thus, the development of new tea products with high levels of these hydrolyzable tannins may provide consumers with additional health benefits beyond those typically associated with Japanese tea. *Camellia taliensis*, the source of the high hydrolyzable tannin trait, is a wild tea relative that lacks the characteristics suitable for commercial tea cultivation in Japan. To address this, breeders developed ‘MK5601’, with a more desirable bush shape and green new shoots. To enhance its versatility, it has been crossed with elite tea cultivars such as ‘Saeakari’, which has excellent tea quality, and ‘Fushun’, which has excellent cold resistance (Yamaguchi *et al.* 1992, Yoshida *et al.* 2012).

Tannins are important not only for their health-promoting effects, but also for the quality of tea, as they contribute to its bitterness and astringency (Yu *et al.* 2014, Zhang *et al.* 2024). The biosynthesis of hydrolyzable tannins is closely related to that of catechins, as they share a common intermediate and both involve the transfer and hydrolysis of the gallate group (Wang *et al.* 2024) (Fig. 1). The enrichment of hydrolyzable tannins may be associated with changes in the overall tannin composition of tea, which could alter quality. However, the genetic mechanism governing the accumulation of hydrolyzable tannins such as theogallin and G-strictinin, as well as their effects on tea quality, has not been fully elucidated. Here, we sought QTLs associated with the contents of hydrolyzable tannins using a linkage map of genome-wide SSR markers. We expect our findings to help elucidate the molecular mechanism controlling tannin accumulation and facilitate the genetic improvement of tea plants.

## Materials and Methods

### Plant materials and DNA extraction

Samples for DNA extraction and HPLC analysis were collected from the Makurazaki Tea Research Station of the National Agriculture and Food Research Organization. QTL analysis was conducted with two F_1_ populations. The first, designated the SM population (*n* = 60), was derived from reciprocal crosses between ‘MK5601’ and ‘Saeakari’. These plants were transplanted to the field in January 2015 after being grown in a greenhouse for one year. The test fields were managed according to standard Japanese tea cultivation methods (Okamoto 2008). Nitrogen fertilizer was applied at 401 kg-N ha^–1^ year^–1^ for mature tea plants. To validate the QTLs, the same analysis was performed on the FM population (*n* = 36), derived from a cross between ‘Fushun’ and ‘MK5601’. ‘MK5601’ was selected for its high theogallin and G-strictinin contents from seedlings of ‘Cha Chuukanbohon Nou 6’, a hybrid of *C. taliensis* and *C. sinensis*. In contrast, ‘Saeakari’ and ‘Fushun’ are common tea cultivars with low hydrolyzable tannin contents. DNA was extracted from leaves with a Plant Genomic DNA Extraction Mini Kit (Favorgen, Ping-Tung, Taiwan).

### HPLC analysis

Samples were prepared as in Kawahara *et al.* (2024). From 10 g of processed tea samples obtained from each cultivar, 6 g was used for sensory evaluation, and the remaining 4 g was used for HPLC analysis. The latter samples were powdered in a CSM-F1 cyclone mill (Shizuoka Seiki, Shizuoka, Japan), and the components of the powders were determined as in Monobe *et al.* (2019). HPLC analyses were performed in 2018 and 2019 on the SM population and corresponding parental lines, and in 2021 on the FM population and corresponding parent. The specific compounds measured by HPLC are shown in Table 1 beside “Raw contents”, classified into several groups according to their chemical structure. In addition, the total amount of substances in each group, referred to as the “Summed contents”, was used in subsequent analyses.

### Linkage map construction

A linkage map of the SM population was constructed using SSR markers developed in previous studies (Hung *et al.* 2008, Jin *et al.* 2006, Liu *et al.* 2017, 2018, Ma *et al.* 2010, 2014, Tan *et al.* 2013, Taniguchi *et al.* 2012, Yao *et al.* 2012), and 2 newly developed markers (Supplemental Table 1). The procedure for SSR marker genotyping followed Nashima *et al.* (2020). Linkage maps based on the polymorphism data were generated in JoinMap v. 4.1 software (Kyazma B.V., Wageningen, Netherlands) with cross-pollination (CP) mode. Genetic distances were calculated using Kosambi’s mapping function. All segregating markers were incorporated in the initial mapping. Subsequently, markers exhibiting distorted segregation (*P* < 0.01) were excluded, if they were unlinked from other markers demonstrating distorted segregation, and the mapping procedure was reiterated. The correspondence between the linkage groups and chromosomes was assessed by aligning SSR marker sequences against the ‘Seimei’ reference genome using BLASTN v. 2.15.0+ in TASUKE+.

### QTL analysis

QTL analysis was performed in MapQTL v. 6 software (Van Ooijen 2009) using the IM (interval mapping) algorithm. The 95% LOD threshold for QTL significance was determined by conducting 1000 permutation tests. Since the distributions of some of the phenotypic data were not normal, we performed the non-parametric Kruskal–Wallis test, which does not rely on the assumption of normality, and used it to further evaluate the results of the map-based QTL analysis. To verify the QTLs identified, we constructed a linkage map of the FM population using the SSR markers that were polymorphic in the SM population. We then performed HPLC analysis on the FM population.

### Statistical analysis

Normality of the data distribution was tested by using the Shapiro.test function in R software, and data with *P* < 0.05 were judged to be non-normally distributed. To understand correlations between traits, we created a Pearson’s correlation matrix heatmap in R using corrplot packages. Chi-squared tests were used to assess genotype separation with the CHISQ.TEST function in Microsoft Excel. Phenotypic differences between the two alleles were analyzed by Wilcoxon rank sum test in R.

### Sensory evaluation of tea quality

The quality of tea products made from each plant in the SM population was evaluated through sensory testing of four items: color of dry leaf, color of liquor, tea aroma, and tea taste. The procedure and the standard of the test were set with reference to Yamaguchi (2006). Each evaluation item of the tea products was rated relative to those of ‘MK5601’ and ‘Saeakari’ on a 20-point scale.

## Results

### Phenotypic analysis of the SM population

**Fig. 2.**
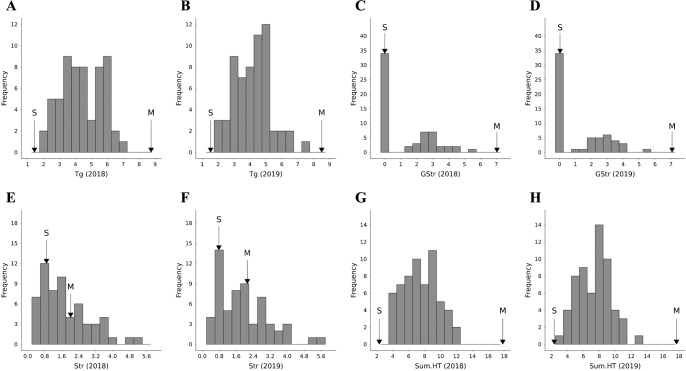
Frequency distributions of hydrolyzable tannin contents in SM population. Trait abbreviations are explained in Table 1. Arrows indicate parental contents (S, ‘Saeakari’; M, ‘MK5601’).

Frequency distributions of hydrolyzable tannin contents are illustrated in Fig. 2 (and of other compounds in Supplemental Figs. 1, 2). The Shapiro–Wilk test of normality showed that the distributions of the theogallin content (Tg) and the total content of hydrolyzable tannins (Sum.HT) were normal, while those of the strictinin content (Str) and the G-strictinin content (GStr) were skewed.

The ranges of Tg were 1.83–6.97 mmol/g DW in 2018 and 1.93–7.40 mmol/g DW in 2019, and were continuously distributed between the parental values (Fig. 2A, 2B). The distribution of GStr was highly skewed (Fig. 2C, 2D). It was barely detectable in 34 seedlings (abbreviated as GStr(–)). In another 26 seedlings (abbreviated as GStr(+)), its content was 1.33–5.71 mmol/g DW in 2018 and 0.925–5.52 mmol/g DW in 2019. The segregation of GStr(+/–) conformed to a 1:1 ratio as determined by the chi-squared test, suggesting that the ability to synthesize G-strictinin is regulated by a single dominant gene in ‘MK5601’ derived from *C. taliensis*. The Str was 0.331–5.14 mmol/g DW in 2018 and 0.352–5.68 mmol/g DW in 2019 (Fig. 2E, 2F). GStr(+) had lower Str than GStr(–).

The Sum.HT was 3.67–11.92 mmol/g DW in 2018 and 3.00–12.51 mmol/g DW in 2019, and was continuously distributed between the parental values (Fig. 2G, 2H). Sum.HT of ‘MK5601’ was 17.82 in 2018 and 17.71 in 2019, clearly higher than in the progeny of ‘MK5601’. Thus, the contents of these compounds were reduced in the genetic background of cultivated teas, and may be regulated along with other common tea polyphenols.

### Correlation of tannin contents in the SM population

**Fig. 3.**
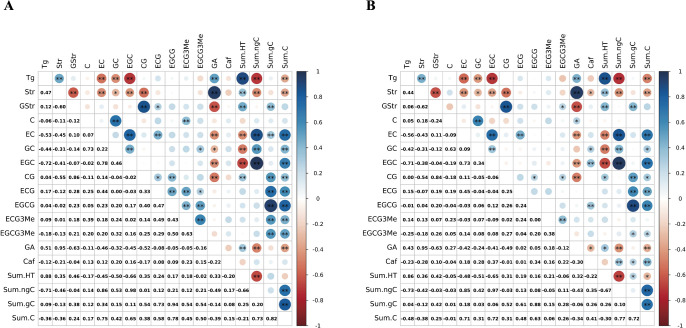
Matrices of Pearson’s correlation coefficients among tannin contents in (A) 2018, (B) 2019. Right side: color key; blue, positive correlations; red, negative correlations. * *P* < 0.05, ** *P* < 0.01.

Since different tannins are synthesized through closely related biosynthesis pathways, we expected their levels to be highly correlated. A matrix of Pearson’s correlation coefficients between compounds showed 11 significant positive correlations (*P* < 0.05) among 20 combinations of the galloylated catechins (CG, ECG, EGCG, ECG3Me, and EGCG3Me) and 6 significant positive correlations among 12 combinations of the non-galloylated catechins (C, EC, GC, and EGC; Fig. 3). There were 4 significant positive correlations between the galloylated and non-galloylated catechins among 20 combinations. These results suggest that the catechins within the same group (galloylated or non-galloylated) likely accumulate through a common biosynthesis pathway in the SM population.

Among the hydrolyzable tannins, Str was correlated positively with Tg but negatively with GStr. GA too was negatively correlated with GStr. This suggests that strictinin and gallic acid are substrates for G-strictinin synthesis. Tg and Str were correlated negatively with EC, GC, and EGC. The Sum.HT was negatively correlated with the total content of the non-galloylated catechins (Sum.ngC), with a correlation coefficient of *r* = –0.66 (*P* < 0.01). The total content of the galloylated catechins (Sum.gC) had a neutral relationship with these two groups. These results suggest a trade-off between the biosynthesis of hydrolyzable tannins and that of non-galloylated catechins.

### Mapping of DNA markers

**Fig. 4.**
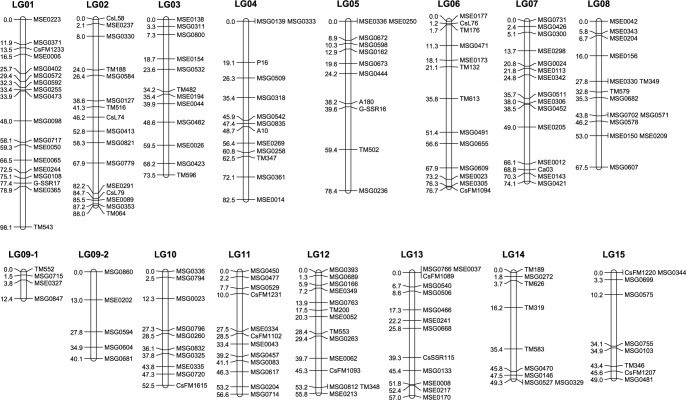
Linkage map of SSR markers in SM population.

A linkage map of the SM population was constructed using 188 DNA markers (Supplemental Table 2). Linkage groups (LGs) containing mapped markers were numbered according to the reference chromosome numbers. Of these markers, 185 (98.4%) were consistently assigned to their corresponding chromosomes (Supplemental Table 2). However, our linkage map comprised 16 LGs, not consistent with the 15 chromosomes of tea, owing to fragmentation of chromosome 9 (Fig. 4). SSR markers on chromosome 9 had distorted segregation (Supplemental Table 3), particularly marker MSG0860, where allele combinations from ‘Z1’ (a seed parent of ‘Saeakari’) and ‘Cha Chuukanbohon Nou 6’ were not observed. The total length of the linkage map was 1011.5 cM, with an average distance of 5.4 cM between adjacent loci. It is close to the 992.4 cM genetic map of ‘Fushun’, a parent of ‘Seimei’ (Chang *et al.* 2017).

### QTLs controlling tannin contents

**Fig. 5.**
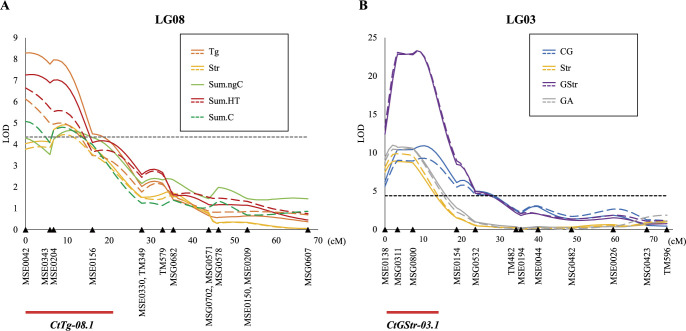
LOD curves obtained by interval mapping in (A) LG08 and (B) LG03 in SM population. Dashed lines, 2018; solid lines, 2019. Dashed horizontal rule, LOD significance threshold (average score of 95% LOD thresholds for each trait). Intervals highlighted in red represent overlapping confidence intervals of QTL clusters.

We mapped QTLs for the contents of tannins (Table 1) on the linkage map (Fig. 4); IM detected 13 putative QTLs in both years for tannin contents on six linkage groups (3, 4, 6, 8, 11, 13; Table 2). Non-parametric Kruskal–Wallis tests confirmed all QTLs, indicating that the results were not affected by the non-normal distribution of certain traits. Among these QTLs, major loci for Tg, Str, Sum.ngC, Sum.HT, and Sum.C were consistently detected in the same region of LG08 in 2018 and 2019 (Fig. 5A), and major loci for CG, Str, GStr, and GA were collocated in the same region of LG03 in both years (Fig. 5B). These results reveal two stable QTL clusters in the population, which we named *CtTg-08.1* and *CtGStr-03.1* (Table 2).

**Fig. 6.**
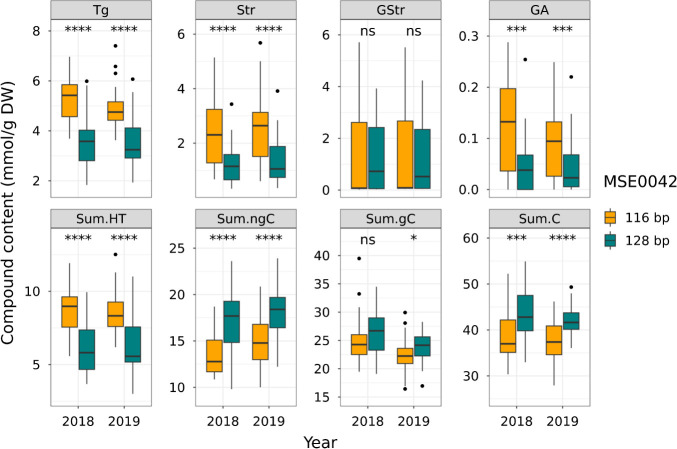
Boxplot of tannin contents among MSE0042 genotypes in SM population. Asterisks indicate significant differences at * *P* < 0.05, ** *P* < 0.01, *** *P* < 0.005, **** *P* < 0.001.

**Fig. 7.**
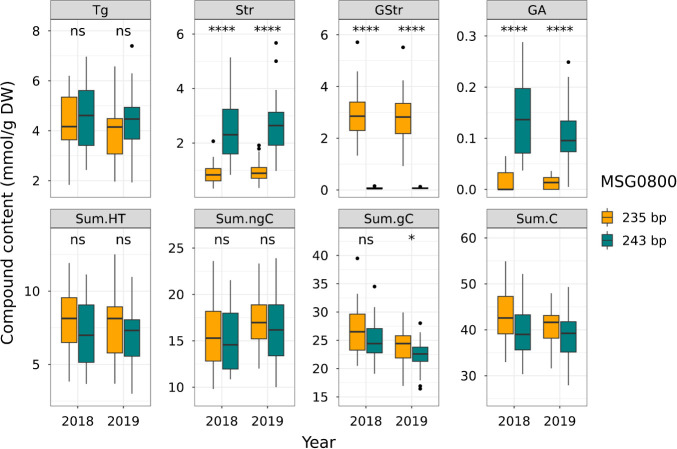
Boxplot of tannin contents among MSG0800 genotypes in SM population. Asterisks indicate significant differences at * *P* < 0.05, **** *P* < 0.001.

The relationships between *CtTg-08.1* and *CtGStr-03.1* genotypes and tannin contents in the SM population are summarized in Figs. 6 and 7. At marker MSE0042, near the *CtTg-08.1* locus, inheritance of a 116-bp allele derived from *C. taliensis* was associated with increased levels of Tg, Str, GA, and Sum.HT, but decreased levels of Sum.ngC, Sum.gC, and Sum.C (Fig. 6). At marker MSG0800, near the *CtGStr-03.1* locus, inheritance of a 235-bp allele derived from *C. taliensis* was associated with increased levels of GStr, Sum.gC, and Sum.C, but decreased levels of Str and GA (Fig. 7). These QTLs appear to have a broad effect on tannin contents and may play an important role in the upstream regulation of tannin biosynthesis. Alignment of the positive or negative regulation of tannin contents associated with these loci with the results of correlation analysis (Fig. 3) suggests that these loci had major effects on tannin contents in the SM population.

Both QTLs were associated with traits Str and GA. Genetic interactions were found between these loci, with the 235-bp allele of MSG0800 suppressing the effects of the 116-bp allele of MSE0042 (Supplemental Fig. 3).

### Validation of QTLs in the FM population

**Fig. 8.**
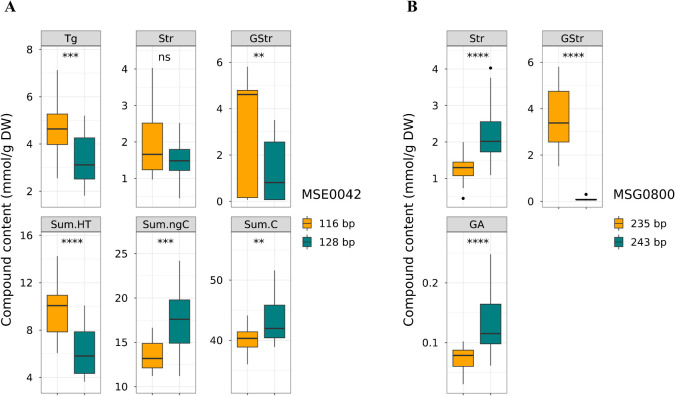
Boxplots showing (A) tannin contents among MSE0042 genotypes and (B) tannin contents among MSG0800 in FM population. Asterisks indicate significant differences at ** *P* < 0.01, *** *P* < 0.005, **** *P* < 0.001.

To verify the effects of the QTL clusters detected within the SM population, we conducted a QTL analysis of the FM population, using 25 SSR markers in LG03 and LG08 to detect QTLs controlling hydrolyzable tannin contents (Supplemental Table 4). As in the SM population, a major QTL for Sum.HT (explaining 42.3% of the variation) was detected in LG08, nearest to the SSR marker MSE0042 (Table 3). At MSE0042, inheritance of a 116-bp allele was associated with increased levels of Tg, GStr, and Sum.HT, but decreased levels of Sum.ngC and Sum.C (Fig. 8A). At *CtTg-08.1*, a significant QTL was detected only for Sum.HT (Table 3), which may be due primarily to reduced statistical power resulting from the smaller sample size of the FM population (*n* = 36) than of the SM population (*n* = 60). Despite this constraint, significant differences were revealed in multiple traits by MSE0042 genotype analysis, supporting the existence of pleiotropic effects of *CtTg-08.1* even in the FM population.

In LG03, a QTL cluster for CG, Str, GStr, and GA was detected. A major QTL for GStr, explaining 76.8% of the phenotypic variation, was detected, nearest to SSR marker MSG0800 (Table 3). At MSG0800, inheritance of a 235-bp allele was associated with increased levels of GStr, but decreased levels of Str and GA (Fig. 8B). These results confirm the pleiotropic effects of QTL clusters *CtTg-08.1* and *CtGStr-03.1* in different genetic backgrounds.

### Effects of *CtTg-08.1* and *CtGStr-03.1* on tea quality

Hydrolyzable tannin contents in common tea cultivars are typically low, and their effects on tea quality remain unknown. The results of sensory testing showed no correlation between the two detected QTL clusters and tea quality except for dry leaf color (Table 4). MSE0042 genotypes significantly affected dry leaf color: cultivars with higher hydrolyzable tannin contents were less green and were perceived as inferior to those with low contents. In the FM population, we also found a negative correlation between the metric hue angle of dry leaf and its hydrolyzable tannin and theogallin contents (Supplemental Fig. 4). It remains unclear whether these color changes are directly related to alterations in tannin composition or result from the action of nearby genes.

## Discussion

### Pathway of hydrolyzable tannin accumulation

The correlation matrix of tannin contents in the SM population (Fig. 3) revealed numerous positive correlations between similar compounds in non-galloylated and galloylated catechins. These results differ from those obtained using albino half-sibs (Chen *et al.* 2022), possibly because of differences in the genetic backgrounds of the populations used and the inclusion of hydrolyzable tannins in the analysis.

All hydrolyzable tannins analyzed in this study are based on gallic acid. Gallic acid biosynthesis in *C. sinensis* involves the oxidation of 3-dehydroshikimate, using the coenzyme NADP+ as a substrate, likely by the product of *3-dehydroquinate dehydratase / shikimate dehydrogenase (DQD/SDH)* (Huang *et al.* 2019). CsDQD/SDH reduces 3-dehydroquinate to produce shikimic acid, an early precursor of the phenylpropanoid pathway, the rate-limiting step in flavonoid biosynthesis (Fig. 1). Thus, gallic acid biosynthesis and flavonoid biosynthesis may compete in this DQD/SDH-catalyzed step. The significant negative correlation between hydrolyzable tannins and non-galloylated catechins in the SM population’s tannin composition (Fig. 3) may strongly reflect the influence of such redox reactions upstream of the shikimate pathway. Meanwhile, galloylated catechins were neutral with respect to hydrolyzable tannins and non-galloylated catechins, likely owing to their chemical properties as both gallic acid derivatives and flavonoid compounds (Fig. 1).

The trade-off between the contents of hydrolyzable tannins and non-galloylated catechins is caused not only by genetic differences but also by cultivation methods. For example, light shading, used to improve green tea quality, increases hydrolyzable tannin contents, especially that of theogallin, while decreasing catechin contents, particularly non-galloylated (Ji *et al.* 2018, Matsunaga *et al.* 2016, Wang *et al.* 2012). This trade-off is also related to the downregulation of the gene for phenylalanine ammonia-lyase, which plays an important role upstream of flavonoid biosynthesis, under light shading (Wang *et al.* 2012). Therefore, the *C. taliensis*-type allele of *CtTg-08.1*, detected as a QTL that increases hydrolyzable tannin contents and decreases catechin contents (especially non-galloylated), may also inhibit flavonoid biosynthesis. Further fine mapping is necessary to identify the candidate genes that contribute to the high hydrolyzable tannin traits.

Common tea cultivars have almost no G-strictinin, but the inheritance of an allele from *C. taliensis* in the *CtGStr-03.1* region conferred the ability to synthesize it. In *C. oleifera*, hydrolyzable tannin synthesis is catalyzed by serine carboxypeptidase-like acyltransferases (SCPL-ATs) (Wang *et al.* 2024). In tea plants, several important enzymes involved in galloylation have been identified, including that encoded by *CsUGT84A22* (Cui *et al.* 2016), which catalyzes gallic acid glycosylation, and those encoded by *CsSCPL4* and *CsSCPL5* (Yao *et al.* 2022), which catalyze catechin galloylation. These genes are densely located on chromosome 3, with a known physical distance of 4.57 Mb from *CsUGT84A22* to *CsSCPL4,5* (Zhao *et al.* 2023). BLAST analysis of SSR marker sequences against the reference genome revealed that *CtGStr-03.1* encompasses these genes (Table 2, Supplemental Tables 2, 5). While *SCPL-AT* genes such as *SCPL4-1* and *SCPL4-2*, which are located in this cluster and whose products catalyze galloylation, are conserved among *Camellia* species, their substrate specificities differ (Zhao *et al.* 2023). Therefore, any of the SCPL-ATs from *C. taliensis* encoded in this region might be able to catalyze strictinin galloylation.

### Use of *CtTg-08.1* and *CtGStr-03.1* in breeding

Tannins play a crucial role in both tea quality and health benefits. Among the catechins, which are the main tannins in tea, galloylated catechins such as epicatechin-gallate and epigallocatechin-gallate have higher antioxidant activity than non-galloylated catechins (Colon and Nerín 2016) but tend to be more bitter and unpleasant tasting (Narukawa *et al.* 2010, Rossetti *et al.* 2009). Such a high concentration of functional ingredients that compromise flavor preference is undesirable in tea products. The effect of hydrolyzable tannins on tea flavor should be analyzed alongside their health-promoting effects.

Theogallin and strictinin have a strong positive correlation with the sweet aftertaste of green tea (Han *et al.* 2022). Theogallin also enhances the umami intensity of matcha (Kaneko *et al.* 2006). However, theogallin itself is an astringent compound, with a low taste threshold (Zhang *et al.* 2024), raising concerns about its negative influence. Nevertheless, the accumulation of theogallin and strictinin does not compromise tea taste (Table 4). While theogallin may contribute to astringency, its synthesis appears to be balanced by non-galloylated catechin contents (Fig. 6), leaving bitterness and astringency unchanged. However, the high-hydrolyzable-tannin cultivars had inferior dry leaf color to the low-hydrolyzable-tannin cultivars (Table 4, Supplemental Fig. 4). In addition to chlorophyll content and chlorophyll-*a/b* ratio, the rate of conversion of chlorophyll to pheophytin during the tea manufacturing process and the intracellular pH involved in this reaction are important to dry leaf color (Hirono *et al.* 2024, Ijichi and Tokuda 2015). Our results suggest that *CtTg-08.1* is associated with such determinants of color loss. Dry leaf color is a more crucial trait in matcha and powdered tea, which are popular. But there was no association between *CtGStr-03.1* allele type and tea quality. Therefore, high accumulation of hydrolyzable tannins such as theogallin and G-strictinin can yield health-promoting effects different from those of common tea polyphenols without adversely affecting tea flavor such as aroma and taste.

Using markers associated with the two QTL clusters identified here, it is possible to select cultivars with high hydrolyzable tannin contents from the progeny of ‘MK5601’, but at a lower level than in the parent (Fig. 2G, 2H). This reduction is likely due to differences in the background genes related to tannin biosynthesis in cultivated tea; for example, *C. taliensis* has a lower total catechin content than *C. sinensis* (Gao *et al.* 2008). A future challenge is to establish breeding methods that maintain theogallin or G-strictinin content while improving the practical agronomic traits of tea. In future research, we will fine-map *CtTg-08.1* and identify the role that the underlying genes play in the biosynthesis and metabolism of tannins, aiming to improve the efficiency of developing new tea cultivars with good health-promoting effects. This study is an important first step toward developing such new tea products.

## Author Contribution Statement

KT, SY and AO prepared the tea samples; MM, KE and SN carried out HPLC measurements; KT, FT and AO carried out genetic analysis; KY, SY, MM and SN carried out the sensory test; and KT and FT wrote the manuscript, with assistance from the other authors.

## Supplementary Material

Supplemental Figures

Supplemental Tables

## Figures and Tables

**Table 1.** List of compounds measured by HPLC

Content	Classification	Trait abbrev.	Trait description (mmol/g DW)
Raw content	Hydrolyzable tannins	Tg	Theogallin content
		Str	Strictinin content
		GStr	G-strictinin content
	Non-galloylated catechins	C	(+)-Catechin content
		EC	(–)-Epicatechin content
		GC	(–)-Gallocatechin content
		EGC	(–)-Epigallocatechin content
	Galloylated catechins	CG	(+)-Catechin gallate content
		ECG	(–)-Epicatechin gallate content
		EGCG	(–)-Epigallocatechin gallate content
		ECG3Me	Epicatechin-3-O-(3-O-methyl) gallate content
		EGCG3Me	Epigallocatechin-3-O-(3-O-methyl) gallate content
	Others	GA	Gallic acid content
		Caf	Caffeine content
Summed content	–	Sum.HT	Total amount of hydrolyzable tannins
		Sum.ngC	Total amount of non-galloylated catehins
		Sum.gC	Total amount of galloylated catechins
		Sum.C	Total amount of catechins

**Table 2.** QTLs detected in SM population

Year	Linkage group	QTL cluster name	Trait	LOD peak	Peak position (cM)	Nearest marker	1-LOD interval (cM)	Effect (%)*^a^*	*P*-value*^b^*
2018	LG03	*CtGStr-03.1*	Str	8.92	3.0	MSG0311	0.49–8.5	49.6	<0.0001
			GStr	23.30	8.3	MSG0800	3.0–10.4	83.3	<0.0001
			CG	10.91	10.3	MSG0800	2.8–13.4	56.7	<0.0001
			GA	10.98	2.0	MSG0311	0.58–8.4	56.9	<0.0001
	LG04		C	8.47	36.4	MSG0318	33.4–39.8	47.8	<0.0001
	LG06		EGCG3Me	9.51	8.7	MSG0471	5.2–13.0	51.8	<0.0001
	LG08	*CtTg-08.1*	Tg	8.3	1.0	MSE0042	0.0–10.8	47.1	<0.0001
			Str	4.93	9.7	MSE0204	0.0–15.6	31.5	<0.001
			Sum.HT	7.28	1.0	MSE0042	0.0–11.5	42.8	<0.0001
			Sum.ngC	4.66	10.7	MSE0204	6.0–21.0	30.1	<0.001
	LG11		ECG	4.73	56.6	MSG0714	48.1–56.6	30.4	<0.0005
			ECG3Me	4.31	53.2	MSG0204	49.8–56.6	28.2	<0.0001
	LG13		C	4.72	22.2	MSE0241	8.5–29.3	30.4	<0.0005
2019	LG03	*CtGStr-03.1*	Str	9.90	3.0	MSG0311	0.81–8.3	53.2	<0.0001
			GStr	23.20	8.3	MSG0800	2.7–10.5	83.2	<0.0001
			CG	9.28	10.3	MSG0800	2.3–14.0	51.0	<0.0001
			GA	10.56	7.3	MSG0800	0.66–8.8	55.5	<0.0001
	LG04		C	8.13	36.4	MSG0318	33.1–39.9	46.4	<0.0001
	LG06		EGCG3Me	11.55	8.7	MSG0471	6.1–15.4	58.8	<0.0001
	LG08	*CtTg-08.1*	Tg	6.13	0.0	MSE0042	0.0–5.2	37.5	<0.0001
			Str	4.47	9.7	MSE0204	0.0–16.5	27.6	<0.005
			Sum.HT	6.65	0.0	MSE0042	0.0–6.1	40.0	<0.0001
			Sum.C	5.08	0.0	MSE0042	0.0–15.4	32.3	<0.0005
	LG11		ECG	5.08	34.4	MSE0043	29.1–41.3	32.3	<0.001
			ECG3Me	4.92	53.2	MSG0204	50.0–56.6	31.5	<0.0001
	LG13		GC	5.25	23.2	MSE0241	18.6–30.5	33.2	<0.0005

*^a^* Percentage of phenotypic variance explained by the QTL. *^b^* Kruskal-Wallis test results.

**Table 3.** QTLs detected in FM population

Linkage group	QTL cluster name	Trait	LOD peak	Peak position (cM)	Nearest marker	Effect (%)*^a^*	*P*-value*^b^*
LG08	*CtTg-08.1*	Sum.HT	4.29	0.0	MSE0042	42.3	<0.005
LG03	*CtGStr-03.1*	Str	4.20	8.4	MSE0154	41.6	<0.001
		GStr	11.43	6.4	MSG0800	76.8	<0.0001
		CG	9.36	7.1	MSG0800	69.8	<0.0001
		GA	3.81	6.4	MSG0800	38.6	<0.005

*^a^* Percentage of phenotypic variance explained by the QTL. *^b^* Kruskal-Wallis test results.

**Table 4.** Differences in tea quality among genotypes of markers in QTL clusters

Marker	Allele	Tea quality evaluated through sensory testing *^a^*			
		Color of dry leaf	Tea aroma	Color of liquor	Tea taste
MSE0042	128 bp	11.2	11.7	10.4	9.8
	116 bp	9.7**	11.4	9.9	10.2
MSG0800	243 bp	10.3	11.5	10.0	10.1
	235 bp	10.6	11.6	10.4	9.8

*^a^* Asterisks indicate significant differences between genotypes at ** *P* < 0.01.
