# Supplementary material for: Identification of QTLs controlling hydrolyzable tannin contents derived from a wild tea relative, Camellia taliensis
Source: Breed Sci. 2025 Jul 31;75(4):255–66. doi: 10.1270/jsbbs.24079 (PMC13051635; doi:10.1270/jsbbs.24079)
Supplement: Supplementary file 1 — Supplemental Figures [file 75_255_s1.pdf]

doi: 10.1270/jsbbs.24079

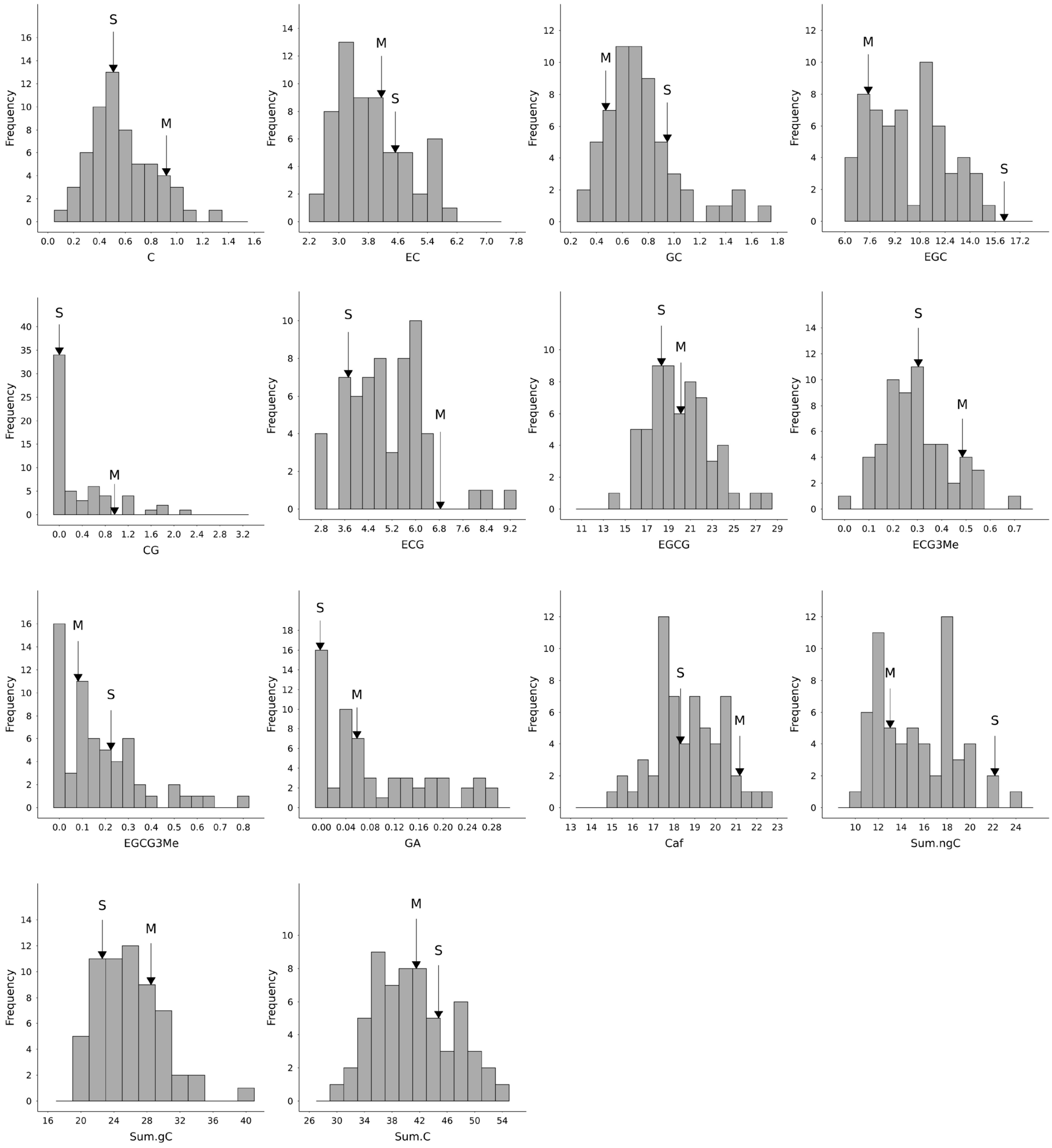

Supplemental Figure 1. Frequency distributions of tannin and other contents in SM population in 2018. Trait abbreviations are explained in Table 1. Arrows indicate parental contents (S, 'Saeakari'; M, 'MK5601').

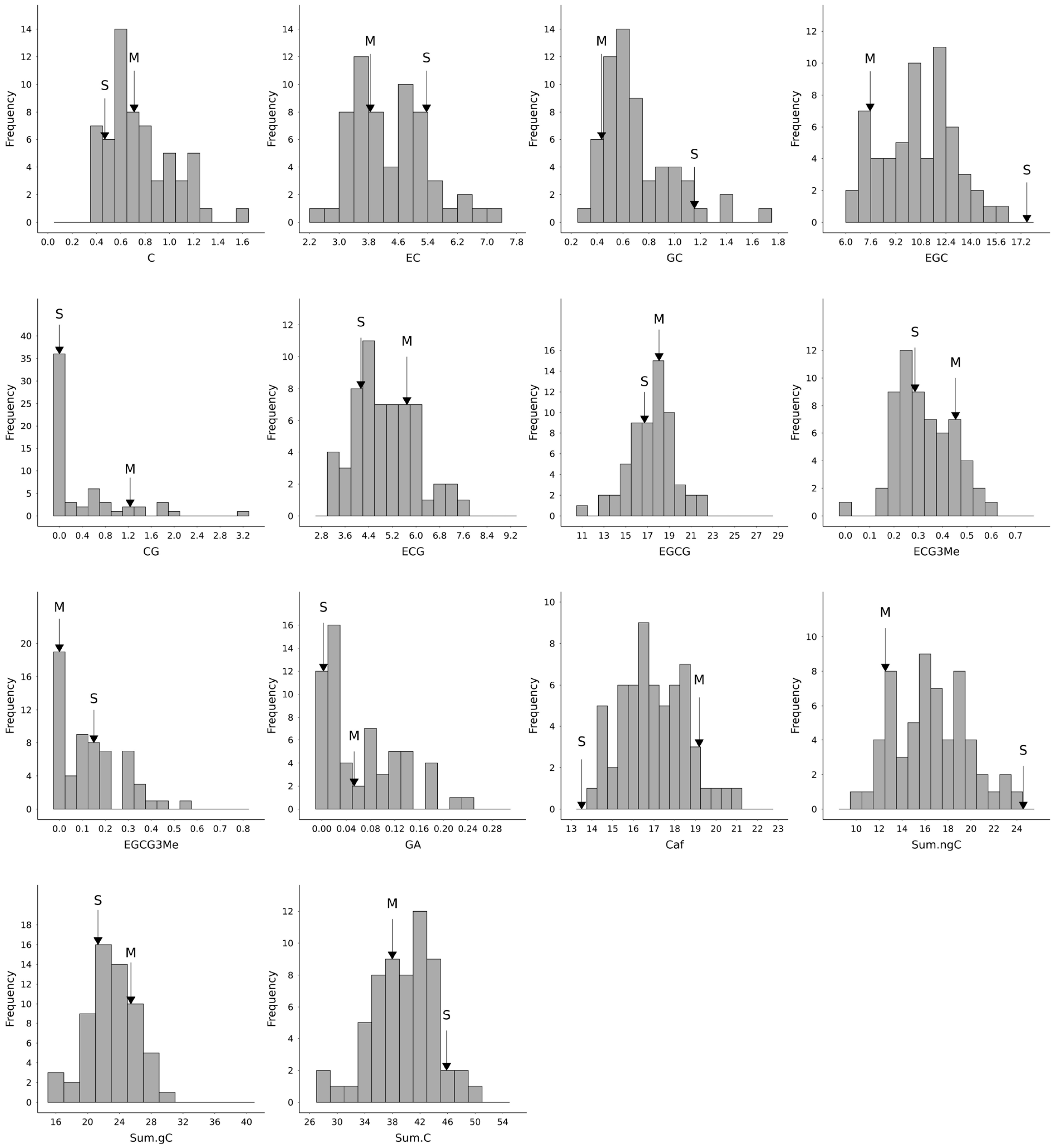

Supplemental Figure 2. Frequency distribution of tannin and other contents in SM population in 2019. Trait abbreviations are explained in Table 1. Arrows indicate parental contents (S, ‘Saeakari’; M, ‘MK5601’).

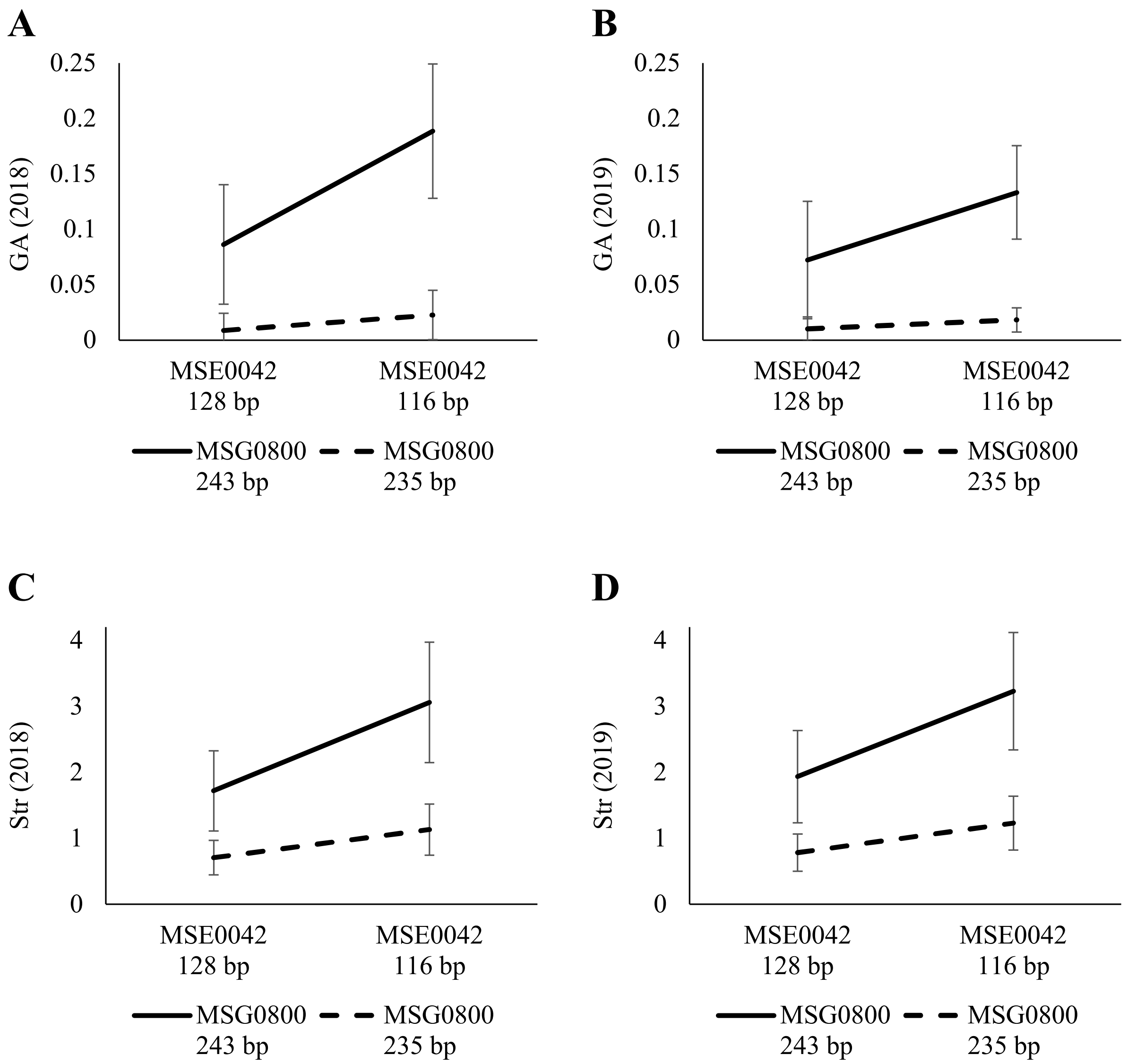

Supplemental Figure 3. Interaction plot for markers MSE0042 and MSG0800: (A) GA in 2018; (B) GA in 2019; (C) Str in 2018; and (D) Str in 2019.

A

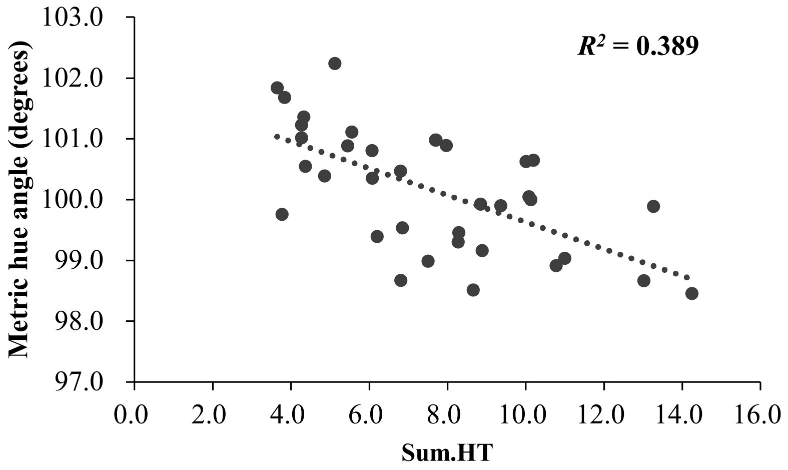

B

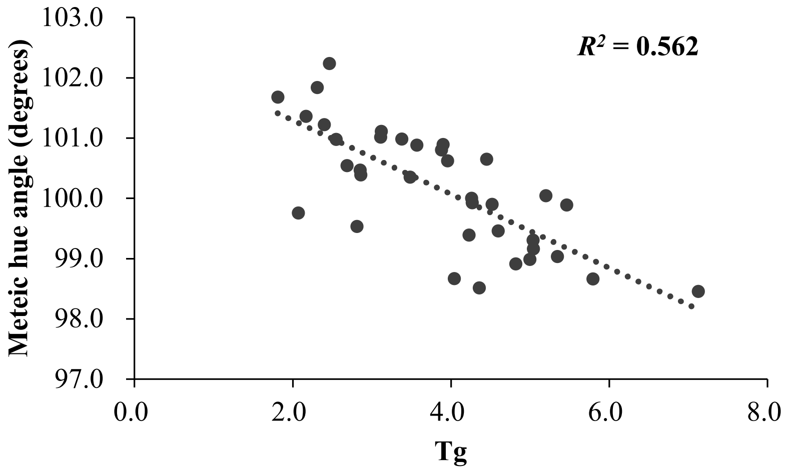

Supplemental Figure 4. Scatter plots of metric hue angle against hydrolyzable tannin contents in FM population: (A) Sum.HT; and (B) Tg.
