# Supplementary material for: Identification of QTLs controlling hydrolyzable tannin contents derived from a wild tea relative, Camellia taliensis
Source: Breed Sci. 2025 Jul 31;75(4):255–66. doi: 10.1270/jsbbs.24079 (PMC13051635; doi:10.1270/jsbbs.24079)
Supplement: Supplementary file 2 — Supplemental Tables [file 75_255_s2.pdf]

doi: 10.1270/jsbbs.24079

Supplemental Table 1. Newly developed SSR markers.

Marker name	Motif	Forward primer	Reverse primer	Product size (bp)
MSG0847	(GA) 10	TAGCGACGGGGTAAAGCAAT	AGCTAGCCTCTCTTGGTCAGA	249
MSE0860	(AG) 19	TGGCAAGAGTTGTGCCATGT	TGTAACTGAAAAACGAAAAAGGCA	197

Supplemental Table 2. Summary of marker sets validated in SM population.

Marker name	Linkage group	Position (cM)	Physical position (Mbp)	Reference	Segregation [b]
MSE0223	LG01	0.00	3.3	Taniguchi et al., 2012	<ab × cd>
MSG0371	LG01	11.86	26.7	Taniguchi et al., 2012	<nn × np>
CsFM1233	LG01	13.54	23.1	Tan et al., 2013	<ab × cd>
MSE0006	LG01	16.53	37.6	Taniguchi et al., 2012	<nn × np>
MSG0402	LG01	25.68	67.8	Taniguchi et al., 2012	<nn × np>
MSG0572	LG01	29.36	93.8	Taniguchi et al., 2012	<ef × eg>
MSG0592	LG01	32.32	103.2	Taniguchi et al., 2012	<ab × cd>
MSG0255	LG01	33.45	121.9	Taniguchi et al., 2012	<nn × np>
MSG0473	LG01	33.86	119.7	Taniguchi et al., 2012	<ef × eg>
MSG0098	LG01	47.96	150.8	Taniguchi et al., 2012	<ef × eg>
MSG0717	LG01	58.07	181.6	Taniguchi et al., 2012	<ab × cd>
MSE0050	LG01	59.33	183.8	Taniguchi et al., 2012	<nn × np>
MSE0065	LG01	66.53	199.4	Taniguchi et al., 2012	<ab × cd>
MSE0244	LG01	72.54	214.4	Taniguchi et al., 2012	<ef × eg>
MSG0108	LG01	75.06	224.4	Taniguchi et al., 2012	<ab × cd>
G-SSR17	LG01	77.37	225.3	Liu et al., 2017	<nn × np>
MSE0365	LG01	78.94	242.1	Taniguchi et al., 2012	<lm × ll>
TM543	LG01	98.08	219.0	Ma et al., 2014	<lm × ll>
CsL58	LG02	0.00	1.4	Liu et al., 2018	<nn × np>
MSE0237	LG02	2.14	3.3	Taniguchi et al., 2012	<nn × np>
MSG0330	LG02	7.99	11.8	Taniguchi et al., 2012	<nn × np>
TM188	LG02	24.03	37.9	Yao et al., 2012	<nn × np>
MSG0584	LG02	26.37	51.7	Taniguchi et al., 2012	<ef × eg>
MSG0127	LG02	38.61	69.8	Taniguchi et al., 2012	<ab × cd>
TM516	LG02	41.28	80.6	Ma et al., 2014	<nn × np>
CsL74	LG02	46.25	94.9	Liu et al., 2018	<nn × np>
MSG0413	LG02	52.79	120.1	Taniguchi et al., 2012	<nn × np>
MSG0821	LG02	58.33	147.9	Taniguchi et al., 2012	<ab × cd>
MSG0779	LG02	67.88	172.0	Taniguchi et al., 2012	<hk × hk>
MSE0291	LG02	82.18	198.0	Taniguchi et al., 2012	<ef × eg>
CsL79	LG02	84.69	205.6	Liu et al., 2018	<ef × eg>
MSE0089	LG02	85.51	208.4	Taniguchi et al., 2012	<ef × eg>
MSG0353	LG02	87.16	216.6	Taniguchi et al., 2012	<ab × cd>
TM064	LG02	88.03	218.0	Ma et al., 2010	<hk × hk>
MSE0138	LG03	0.00	18.6	Taniguchi et al., 2012	<nn × np>
MSG0311	LG03	3.27	30.1	Taniguchi et al., 2012	<ef × eg>
MSG0800	LG03	7.25	40.3	Taniguchi et al., 2012	<ef × eg>
MSE0154	LG03	18.68	62.2	Taniguchi et al., 2012	<ab × cd>
MSG0532	LG03	23.57	68.9	Taniguchi et al., 2012	<ab × cd>

Marker name	Linkage group	Position (cM)	Physical position (Mbp)	Reference	Segregation [b]
TM482	LG03	34.17	77.5	Ma et al., 2014	<lm × ll>
MSE0194	LG03	35.43	101.5	Taniguchi et al., 2012	<ef × eg>
MSE0044	LG03	39.89	113.0	Taniguchi et al., 2012	<ab × cd>
MSG0482	LG03	48.59	153.6	Taniguchi et al., 2012	<ab × cd>
MSE0026	LG03	59.48	183.2	Taniguchi et al., 2012	<ab × cd>
MSG0423	LG03	68.23	200.5	Taniguchi et al., 2012	<ab × cd>
TM596	LG03	73.45	216.3	Ma et al., 2014	<ab × cd>
MSG0139	LG04	0.00	29.4	Taniguchi et al., 2012	<nn × np>
MSG0333	LG04	0.04	2.1	Taniguchi et al., 2012	<ab × cd>
P16	LG04	19.09	51.9	Jin et al., 2006	<lm × ll>
MSG0509	LG04	26.35	70.0	Taniguchi et al., 2012	<ef × eg>
MSG0318	LG04	35.41	95.5	Taniguchi et al., 2012	<ef × eg>
MSG0542	LG04	45.94	158.7	Taniguchi et al., 2012	<ab × cd>
MSG0835	LG04	47.42	155.8	Taniguchi et al., 2012	<ef × eg>
A10	LG04	48.74	172.9	Tan et al., 2013	<ab × cd>
MSE0269	LG04	56.45	195.5	Taniguchi et al., 2012	<ef × eg>
MSG0258	LG04	60.76	205.7	Taniguchi et al., 2012	<ef × eg>
TM347	LG04	62.48	207.6	Ma et al., 2014	<ef × eg>
MSG0361	LG04	72.05	55.3	Taniguchi et al., 2012	<ab × cd>
MSE0014	LG04	82.48	244.3	Taniguchi et al., 2012	<ab × cd>
MSE0250	LG05	0.00	8.2	Taniguchi et al., 2012	<ab × cd>
MSE0336	LG05	0.00	10.3	Taniguchi et al., 2012	<ab × cd>
MSG0672	LG05	8.90	174.8 (Chr13)	Taniguchi et al., 2012	<lm × ll>
MSG0598	LG05	10.31	36.1	Taniguchi et al., 2012	<ab × cd>
MSG0162	LG05	12.90	52.1	Taniguchi et al., 2012	<lm × ll>
MSG0673	LG05	19.57	89.4	Taniguchi et al., 2012	<lm × ll>
MSG0444	LG05	24.22	104.7	Taniguchi et al., 2012	<lm × ll>
A180	LG05	38.17	173.7	Tan et al., 2013	<lm × ll>
G-SSR16	LG05	39.62	176.5	Liu et al., 2017	<ab × cd>
TM502	LG05	59.35	209.1	Ma et al., 2014	<lm × ll>
MSG0236	LG05	78.41	124.0	Taniguchi et al., 2012	<nn × np>
MSE0177	LG06	0.00	5.2	Taniguchi et al., 2012	<nn × np>
CsL76	LG06	1.20	14.4	Liu et al., 2018	<ef × eg>
TM176	LG06	1.70	17.5	Yao et al., 2012	<nn × np>
MSG0471	LG06	11.35	39.3	Taniguchi et al., 2012	<ab × cd>
MSE0173	LG06	18.11	59.1	Taniguchi et al., 2012	<ab × cd>
TM132	LG06	21.15	67.8	Yao et al., 2012	<nn × np>
TM613	LG06	35.80	94.0	Ma et al., 2014	<ef × eg>
MSG0491	LG06	51.43	126.1	Taniguchi et al., 2012	<ab × cd>
MSG0655	LG06	56.58	140.3	Taniguchi et al., 2012	<lm × ll>
MSG0609	LG06	67.88	179.1	Taniguchi et al., 2012	<ef × eg>

Marker name	Linkage group	Position (cM)	Physical position (Mbp)	Reference	Segregation [b]
MSE0023	LG06	73.23	195.8	Taniguchi et al., 2012	<nn × np>
MSE0305	LG06	76.26	209.5	Taniguchi et al., 2012	<nn × np>
CsFM1094	LG06	76.67	213.0	Tan et al., 2013	<ab × cd>
MSG0731	LG07	0.00	18.9	Taniguchi et al., 2012	<nn × np>
MSG0426	LG07	2.38	39.4	Taniguchi et al., 2012	<ef × eg>
MSG0300	LG07	5.09	45.4	Taniguchi et al., 2012	<ef × eg>
MSE0298	LG07	13.71	58.1	Taniguchi et al., 2012	<hk × hk>
MSG0024	LG07	20.84	67.7	Taniguchi et al., 2012	<ef × eg>
MSE0113	LG07	21.79	72.4	Taniguchi et al., 2012	<lm × ll>
MSE0342	LG07	24.79	81.5	Taniguchi et al., 2012	<ab × cd>
MSG0511	LG07	35.73	97.2	Taniguchi et al., 2012	<nn × np>
MSE0306	LG07	38.00	133.5	Taniguchi et al., 2012	<lm × ll>
MSG0452	LG07	38.50	128.8	Taniguchi et al., 2012	<ef × eg>
MSE0205	LG07	49.01	168.0	Taniguchi et al., 2012	<ab × cd>
MSE0012	LG07	66.09	188.5	Taniguchi et al., 2012	<ab × cd>
Ca03	LG07	68.76	195.1	Hung et al., 2008	<lm × ll>
MSE0143	LG07	70.27	206.6	Taniguchi et al., 2012	<nn × np>
MSG0421	LG07	74.14	209.5	Taniguchi et al., 2012	<ef × eg>
MSE0042	LG08	0.00	8.3	Taniguchi et al., 2012	<ab × cd>
MSE0343	LG08	5.82	29.2	Taniguchi et al., 2012	<ef × eg>
MSE0204	LG08	6.68	33.6	Taniguchi et al., 2012	<ab × cd>
MSE0156	LG08	15.98	40.8	Taniguchi et al., 2012	<ef × eg>
MSE0330	LG08	27.79	60.4	Taniguchi et al., 2012	<nn × np>
TM349	LG08	27.79	67.3	Ma et al., 2014	<nn × np>
TM579	LG08	32.78	92.7	Ma et al., 2014	<nn × np>
MSG0682	LG08	35.33	102.6	Taniguchi et al., 2012	<ef × eg>
MSG0571	LG08	43.81	152.3	Taniguchi et al., 2012	<ef × eg>
MSG0702	LG08	43.81	157.5	Taniguchi et al., 2012	<ab × cd>
MSG0578	LG08	46.15	168.5	Taniguchi et al., 2012	<ab × cd>
MSE0209	LG08	53.05	172.1	Taniguchi et al., 2012	<lm × ll>
MSE0150	LG08	53.05	172.1	Taniguchi et al., 2012	<lm × ll>
MSG0607	LG08	67.54	130.5 (Chr12)	Taniguchi et al., 2012	<ef × eg>
TM552	LG09-1	0.00	1.7	Ma et al., 2014	<nn × np>
MSG0715	LG09-1	1.50	11.8	Taniguchi et al., 2012	<ab × cd>
MSE0327	LG09-1	3.76	15.2	Taniguchi et al., 2012	<ef × eg>
MSG0847[a]	LG09-1	12.45	28.4	-	<ef × eg>
MSG0860[a]	LG09-2	0.00	54.1	-	<ab × cd>
MSE0202	LG09-2	12.97	92.3	Taniguchi et al., 2012	<ef × eg>
MSG0594	LG09-2	27.79	113.3	Taniguchi et al., 2012	<ef × eg>
MSG0604	LG09-2	34.92	132.0	Taniguchi et al., 2012	<ab × cd>
MSG0681	LG09-2	40.15	163.1	Taniguchi et al., 2012	<ab × cd>

Marker name	Linkage group	Position (cM)	Physical position (Mbp)	Reference	Segregation [b]
MSG0336	LG10	0.00	19.5	Taniguchi et al., 2012	<ab × cd>
MSG0794	LG10	2.54	29.6	Taniguchi et al., 2012	<lm × ll>
MSG0023	LG10	12.27	65.4	Taniguchi et al., 2012	<ab × cd>
MSG0796	LG10	27.26	115.3	Taniguchi et al., 2012	<lm × ll>
MSG0260	LG10	28.48	118.3	Taniguchi et al., 2012	<ef × eg>
MSG0832	LG10	36.08	136.9	Taniguchi et al., 2012	<ab × cd>
MSG0325	LG10	37.75	139.2	Taniguchi et al., 2012	<nn × np>
MSE0335	LG10	43.83	158.1	Taniguchi et al., 2012	<ab × cd>
MSG0720	LG10	47.32	92.0 (Chr2)	Taniguchi et al., 2012	<ab × cd>
CsFM1615	LG10	52.47	176.1	Tan et al., 2013	<ab × cd>
MSG0450	LG11	0.00	46.2	Taniguchi et al., 2012	<nn × np>
MSG0477	LG11	2.19	62.1	Taniguchi et al., 2012	<ab × cd>
MSG0529	LG11	7.68	68.3	Taniguchi et al., 2012	<ef × eg>
CsFM1231	LG11	10.03	75.8	Tan et al., 2013	<nn × np>
MSE0334	LG11	27.51	91.2	Taniguchi et al., 2012	<ef × eg>
CsFM1102	LG11	28.50	88.2	Tan et al., 2013	<nn × np>
MSE0043	LG11	33.45	102.4	Taniguchi et al., 2012	<ab × cd>
MSG0457	LG11	39.25	122.5	Taniguchi et al., 2012	<nn × np>
MSG0083	LG11	41.08	111.5	Taniguchi et al., 2012	<lm × ll>
MSG0617	LG11	46.26	129.4	Taniguchi et al., 2012	<nn × np>
MSG0204	LG11	53.19	138.7	Taniguchi et al., 2012	<nn × np>
MSG0714	LG11	56.61	147.9	Taniguchi et al., 2012	<nn × np>
MSG0393	LG12	0.00	20.9	Taniguchi et al., 2012	<nn × np>
MSG0689	LG12	1.34	13.1	Taniguchi et al., 2012	<ab × cd>
MSG0166	LG12	5.94	45.9	Taniguchi et al., 2012	<ef × eg>
MSE0349	LG12	7.18	48.8	Taniguchi et al., 2012	<nn × np>
MSG0763	LG12	13.92	60.7	Taniguchi et al., 2012	<ab × cd>
TM200	LG12	17.47	86.9	Yao et al., 2012	<lm × ll>
MSE0052	LG12	20.34	92.6	Taniguchi et al., 2012	<ab × cd>
TM553	LG12	28.35	106.0	Ma et al., 2014	<lm × ll>
MSG0263	LG12	29.42	117.9	Taniguchi et al., 2012	<nn × np>
MSE0062	LG12	39.71	134.9	Taniguchi et al., 2012	<hk × hk>
CsFM1093	LG12	45.25	150.9	Tan et al., 2013	<ef × eg>
MSG0812	LG12	53.23	171.1	Taniguchi et al., 2012	<lm × ll>
TM348	LG12	53.23	171.1	Ma et al., 2014	<lm × ll>
MSE0213	LG12	55.76	171.1	Taniguchi et al., 2012	<ab × cd>
CsFM1089	LG13	0.00	1.2	Tan et al., 2013	<ab × cd>
MSE0037	LG13	0.00	67.2	Taniguchi et al., 2012	<ab × cd>
MSG0766	LG13	0.00	49.6	Taniguchi et al., 2012	<ab × cd>
MSG0540	LG13	6.73	82.0	Taniguchi et al., 2012	<ab × cd>
MSG0506	LG13	8.64	109.4	Taniguchi et al., 2012	<lm × ll>

Marker name	Linkage group	Position (cM)	Physical position (Mbp)	Reference	Segregation [b]
MSG0466	LG13	17.27	107.2	Taniguchi et al., 2012	<ab × cd>
MSE0241	LG13	22.16	129.5	Taniguchi et al., 2012	<nn × np>
MSG0668	LG13	25.79	129.8	Taniguchi et al., 2012	<ab × cd>
CsSSR115	LG13	39.32	150.4	Tan et al., 2013	<hk × hk>
MSG0133	LG13	45.42	162.8	Taniguchi et al., 2012	<ab × cd>
MSE0008	LG13	51.83	185.7	Taniguchi et al., 2012	<lm × ll>
MSE0217	LG13	52.42	165.7	Taniguchi et al., 2012	<nn × np>
MSE0170	LG13	56.99	190.2	Taniguchi et al., 2012	<lm × ll>
TM189	LG14	0.00	0.68	Yao et al., 2012	<nn × np>
MSG0272	LG14	1.80	20.6	Taniguchi et al., 2012	<ef × eg>
TM626	LG14	3.67	43.6	Ma et al., 2014	<lm × ll>
TM319	LG14	16.25	65.0	Ma et al., 2014	<ef × eg>
TM583	LG14	35.41	86.5	Ma et al., 2014	<lm × ll>
MSG0470	LG14	45.79	100.4	Taniguchi et al., 2012	<lm × ll>
MSG0146	LG14	47.55	157.5	Taniguchi et al., 2012	<ef × eg>
MSG0329	LG14	49.28	115.0	Taniguchi et al., 2012	<nn × np>
MSG0527	LG14	49.28	113.7	Taniguchi et al., 2012	<nn × np>
CsFM1220	LG15	0.00	4.9	Tan et al., 2013	<lm × ll>
MSG0344	LG15	0.01	4.3	Taniguchi et al., 2012	<ab × cd>
MSG0699	LG15	3.33	10.9	Taniguchi et al., 2012	<ab × cd>
MSG0575	LG15	10.24	28.4	Taniguchi et al., 2012	<nn × np>
MSG0755	LG15	34.06	63.5	Taniguchi et al., 2012	<ef × eg>
MSG0103	LG15	34.91	64.0	Taniguchi et al., 2012	<ef × eg>
TM346	LG15	43.43	108.4	Ma et al., 2014	<ef × eg>
CsFM1207	LG15	45.61	115.1	Tan et al., 2013	<ab × cd>
MSG0481	LG15	49.00	122.6	Taniguchi et al., 2012	<ef × eg>

[a] Newly developed markers by searching for SSR motifs.

[b] The segregation patterns of genotypes followed the CP mode of JoinMap.

Supplemental Table 3. Chi-squared test of segregation distortion of SSR markers on chromosome 9.

Linkage group	Marker name	Position (cM)	Segregation [a]	Genotype frequenmcy	Expected ratio	χ2	*P*-value
LG09-1	TM552	0.00	<nn × np>	nn(46): np(14)	1:1	17.1	3.6E-05
	MSG0715	1.50	<ab × cd>	ac(30): bc(15): ad(10): bd(5)	1:1:1:1	23.3	1.1E-04
	MSE0327	3.76	<ef × eg>	ee(30): ef(16): eg(10): fg(4)	1:1:1:1	24.8	1.0E-04
	MSG0847	12.45	<ef × eg>	ee(33): ef(16): eg(9): fg(2)	1:1:1:1	35.3	3.2E-06
LG09-2	MSG0860	0.00	<ab × cd>	ac(33): bc(19): ad(8): bd(0)	1:1:1:1	40.9	1.9E-06
	MSE0202	12.97	<ef × eg>	ee(29): ef(20): eg(8): fg(3)	1:1:1:1	27.6	1.2E-04
	MSG0594	27.79	<ef × eg>	ee(23): ef(20): eg(11): fg(6)	1:1:1:1	12.4	1.5E-02
	MSG0604	34.92	<ab × cd>	ac(21): bc(20): ad(11): bd(8)	1:1:1:1	8.4	4.4E-02
	MSG0681	40.15	<ab × cd>	ac(21): bc(14): ad(12): bd(13)	1:1:1:1	3.3	0.12

[a] The segregation patterns of genotypes followed the CP mode of JoinMap.

Supplemental Table 4. Summary of marker sets validated in FM population.

Marker name	Linkage group	Position on the linkage group (cM)	Segregation [a]
MSG0311	LG03	0.00	<ab × cd>
MSE0138	LG03	4.38	<nn × np>
MSG0800	LG03	7.10	<ef × eg>
MSE0154	LG03	8.41	<ef × eg>
MSG0532	LG03	12.51	<ab × cd>
TM482	LG03	19.84	<lm × ll>
MSE0194	LG03	23.01	<ab × cd>
MSE0044	LG03	25.62	<ab × cd>
MSG0420	LG03	37.39	<ab × cd>
MSG0482	LG03	38.95	<hk × hk>
MSG0423	LG03	54.82	<nn × np>
TM596	LG03	55.27	<ab × cd>
MSE0042	LG08	0.00	<ab × cd>
MSE0204	LG08	2.51	<ab × cd>
MSE0343	LG08	2.51	<ab × cd>
MSE0156	LG08	4.12	<nn × np>
TM349	LG08	17.65	<ef × eg>
MSE0330	LG08	20.03	<nn × np>
MSG0682	LG08	28.32	<ef × eg>
TM579	LG08	31.22	<nn × np>
MSG0702	LG08	37.91	<lm × ll>
MSG0571	LG08	38.56	<ef × eg>
MSG0578	LG08	43.41	<ab × cd>
MSE0150	LG08	47.97	<lm × ll>
MSE0209	LG08	47.97	<lm × ll>

[a] The segregation patterns of genotypes followed the CP mode of JoinMap.

Supplemental Table 5. The chromosomal location of genes involved in galloylation.

Gene name	Reference	Gene ID	Physical position (Mbp)
CsUGT84A22	Cui et al., 2016	CsSME03G064800	29.8
CsSCPL4-2	Zhao et al., 2023	CsSME03G079800	33.5
CsSCPL5	Yao et al., 2022	CsSME03G080100	33.8
CsSCPL4-1	Zhao et al., 2023	CsSME03G080800	34.0
